# Acylation of Glucagon-Like Peptide-2: Interaction with Lipid Membranes and *In Vitro* Intestinal Permeability

**DOI:** 10.1371/journal.pone.0109939

**Published:** 2014-10-08

**Authors:** Sofie Trier, Lars Linderoth, Simon Bjerregaard, Thomas Lars Andresen, Ulrik Lytt Rahbek

**Affiliations:** 1 Dept. of Micro- and Nanotechnology, Center for Nanomedicine and Theranostics, Technical University of Denmark, Kgs. Lyngby, Denmark; 2 Diabetes Research Unit, Novo Nordisk, Maaloev, Denmark; Faculdade de Medicina da Universidade de Lisboa, Portugal

## Abstract

**Background:**

Acylation of peptide drugs with fatty acid chains has proven beneficial for prolonging systemic circulation as well as increasing enzymatic stability without disrupting biological potency. Acylation has furthermore been shown to increase interactions with the lipid membranes of mammalian cells. The extent to which such interactions hinder or benefit delivery of acylated peptide drugs across cellular barriers such as the intestinal epithelia is currently unknown. The present study investigates the effect of acylating peptide drugs from a drug delivery perspective.

**Purpose:**

We hypothesize that the membrane interaction is an important parameter for intestinal translocation, which may be used to optimize the acylation chain length for intestinal permeation. This work aims to characterize acylated analogues of the intestinotrophic Glucagon-like peptide-2 by systematically increasing acyl chain length, in order to elucidate its influence on membrane interaction and intestinal cell translocation *in vitro*.

**Results:**

Peptide self-association and binding to both model lipid and cell membranes was found to increase gradually with acyl chain length, whereas translocation across Caco-2 cells depended non-linearly on chain length. Short and medium acyl chains increased translocation compared to the native peptide, but long chain acylation displayed no improvement in translocation. Co-administration of a paracellular absorption enhancer was found to increase translocation irrespective of acyl chain length, whereas a transcellular enhancer displayed increased synergy with the long chain acylation.

**Conclusions:**

These results show that membrane interactions play a prominent role during intestinal translocation of an acylated peptide. Acylation benefits permeation for shorter and medium chains due to increased membrane interactions, however, for longer chains insertion in the membrane becomes dominant and hinders translocation, i.e. the peptides get ‘stuck’ in the cell membrane. Applying a transcellular absorption enhancer increases the dynamics of membrane insertion and detachment by fluidizing the membrane, thus facilitating its effects primarily on membrane associated peptides.

## Introduction

Acylation of peptides with fatty acids is a naturally occurring post-translational modification, which has inspired alteration of therapeutic peptides for drug delivery. Acylation prolongs the systemic circulation half-life of otherwise rapidly cleared peptide drugs, through increased enzymatic stability [1]–[3] and binding to - and piggy-backing on - serum albumin [4]. An additional effect of acylation is increased peptide self-association and aggregation, which has been employed to ensure prolonged release of peptide drugs following subcutaneous injection [5]. Acylation can be performed without disrupting the peptide's biological potency [6], and has been employed for a multitude of therapeutic peptides [2], [4], [7], [8], including several marketed drugs (e.g. insulin and Glucagon-like peptide-1).

The increased enzymatic stability of acylated peptides is particularly beneficial for oral administration, due to the highly metabolic environment in the stomach and intestine [9]. Another requirement for oral drug delivery is adequate absorption through the intestinal epithelial barrier, which is a major challenge for large, hydrophillic peptide drugs [10]. A widely used method for predicting oral absorption *in vivo* is *in vitro* quantification of translocation across monolayers of the human colon cancer cell line (Caco-2), which has been shown to correlate well with oral bioavailability [11], [12]. Acylation has previously been shown to increase intestinal permeability of peptide drugs [6], [7], [13], but detailed investigations of systematic acyl variations are lacking, which would benefit rational new designs of peptide drugs. The *in vitro* intestinal translocation studies can be further supplemented by measurements of peptide binding to model lipid membranes [14]–[16] in order to investigate the influence of membrane binding of acylated peptides on cellular membrane translocation.

Glucagon-like peptide-2 (GLP-2) is a 33 amino acid peptide, which is secreted from the human intestine following nutrient intake [17], [18]. Therapeutically, GLP-2 stimulates intestinal growth and is employed in the treatment of inflammatory bowel diseases (e.g. Crohn's disease) and short bowel syndrome (e.g. following intestinal surgery) [19], [20]. The plasma half-life of GLP-2 in humans is limited to a few minutes [21] due to extensive renal clearance and rapid enzymatic degradation by dipeptidyl peptidase-4 [21], [22]. Furthermore, GLP-2 is presently administered as subcutaneous injections, which compromises patient comfort and compliance, in particular for chronic diseases like Crohn's. It would be highly beneficial to enable oral administration, and the combined effects of prolonged circulation time, improved enzymatic stability and intestinal permeability may render acylated GLP-2 a suitable candidate for oral drug delivery. Currently, however, there are no reports on the intestinal permeability or oral drug delivery potential of acylated GLP-2.

**Figure 1:**
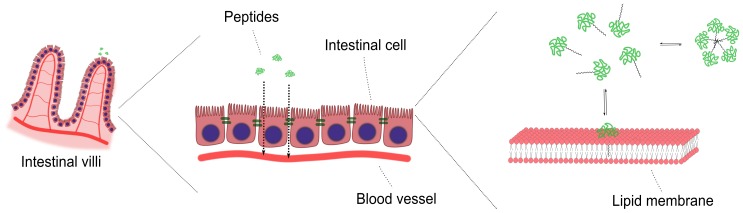
Schematic illustration of the study objective. The membrane interaction and *in vitro* permeability of acylated GLP-2 is investigated using an intestinal cell model and model lipid membranes.

In the present study we synthesized and characterized acylated analogues of GLP-2, with systematically increasing acyl chain length, in order to investigate the effect of the acyl chain on membrane interaction and *in vitro* intestinal permeability. This was achieved by combining investigations of the interaction with lipid membranes and translocation across an intestinal cell model, as outlined in fig. 1.

We hypothesize that the acylation chain length can be optimized for translocation across the intestinal barrier, i.e. a moderate interaction with the lipid cell membrane is beneficial for translocation, whereas a stronger interaction may impair translocation. Acylation is expected to confer membrane affinity to GLP-2, as the native peptide is not membrane active. In this regard, GLP-2 was employed as a model peptide, however, the results may be applicable for development of a rational acylation strategy for other peptide drugs.

Absorption enhancers are often employed to increase oral peptide absorption, which makes it interesting to investigate how these affect the translocation of acylated peptides [23]. In the present study we included two enhancers with different enhancing mechanism, in order to investigate the effect of the enhancing mechanism. Ethylene glycol-bis(*β*-aminoethyl ether)-N, N, N, N-tetraacetic acid (EGTA) is a paracellular enhancer which increases transport between the cells by opening of the tight junctions [24], and sodium dodecyl sulfate (SDS) is a transcellular enhancer which increases transport through the cells at low concentrations, predominantly by fluidizing the cell membrane [25].

We hypothesize that the effect of paracellular enhancers will not be influenced by acylation, whereas the effect of transcellular enhancers that directly interact with the cell membrane may depend on the peptide-membrane interaction, through altered membrane affinity and/or dynamics of membrane insertion.

## Materials and Methods

### Materials

Resin and natural amino acids were purchased at Novabiochem (Germany). c8, c12 and c16 carboxylic acids, Fmoc-beta-Alanine and native GLP-2 were provided by Novo Nordisk A/S. Palmitoyloleoylglycerophosphocholine (POPC) was purchased from Avanti Polar Lipids (USA). HEPES, Ovalbumin (OVA, from chicken egg white) and other standard chemicals were purchased from Sigma-Aldrich (Denmark). DMEM medium, l-glutamine and penicillin/streptomycin was purchased from Lonza (Switzerland). HBSS buffer, fetal bovine serum (FBS), nonessential amino acid and other standard cell culture products were purchased from Gibco (Denmark). Radioactively labeled [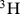]mannitol, scintillation fluid (Microscint-40), luciferase substrate (SteadyLite) and 96-well plates for luciferase assay (CulturPlate, black) were purchased from PerkinElmer (USA). 12 well Transwell plates for Caco-2 cell monolayers (polycarbonate, 12 mm, pore size 0.4 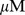) were purchased from Corning Costar Corp. (USA). GLP-2R BHK cells were provided by Novo Nordisk (the cloning was previously described by Thulesen et al. [26] and Sams et al. [27]) and Caco-2 cells (HTB-37) were purchased from ATCC.

### Peptide synthesis

The peptides were synthesized by automated Fmoc based SPPS, using a preloaded Fmoc-Asp(OtBu)-Wang polystyrene LL resin in 0.25 mmol scale on a CEM Liberty microwave peptide synthesizer (CEM Corporation, NC) using standard protocols, with a modified coupling temperature of 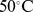 [28]. Fmoc deprotection was carried out in 5% piperidine and 0.05 M HOBt in NMP.

The acylation was conjugated to the lysine side chain by incorporation of lysine as Lys(Mtt), which allowed chemical modification using the standard coupling procedures stated above, following Mtt removal. The Mtt group was removed by washing the resin with DCM and suspending the resin in neat (undiluted) hexafluoroisopropanol for 20 minutes followed by washing with DCM and NMP. After synthesis the resin was washed with DCM, and the peptide was cleaved from the resin by a 3 hour treatment with 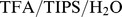 (95/2.5/2.5) followed by precipitation with diethylether. The peptide was dissolved in a suitable solvent (e.g. 1∶1 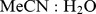) and purified by preparative HPLC on an XBridge c18 column (Waters), using a gradient from 10% MeCN/Buffer (10 mM TRIS and 15 mM (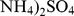, pH 7.3) to 50% MeCN over 40 min, flow rate 60 mL/min. The fractions were analysed by a combination of UPLC and LCMS methods, and the appropriate fractions were pooled and transferred to TFA salt for lyophilization, using a gradient from 10% MeCN/0.1% TFA/MQ to 60% MeCN. The peptides were finally quantified using a chemiluminescent nitrogen detector, as previously described by Fujinari et al. [29].

The isoelectric point of GLP-2 is approximately 4, with a theoretical charge of -4 at neutral pH, and the analogues are expected to be very similar.

### Liposome preparation and characterization

POPC lipid films were prepared by evaporation from chloroform:methanol (9∶1 v/v) using a gentle nitrogen flow. The residual organic solvent was evaporated in vacuum overnight and the lipid films were rehydrated to 25 mM in HEPES buffer (10 mM HEPES, 150 mM NaCl, pH 7.4) at room temperature with frequent vigorous agitation for 1 hour. The multilamellar lipid suspensions were subjected to 10 cycles of freeze-thawing (isopropanol/dry-ice and 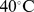 water bath) and extruded to 100 nm liposomes by passing it 21 times through a 100 nm polycarbonate filter in a manual extruder (Avanti Polar Lipids). [30]

The liposome hydrodynamic size after extrusion was measured in a ZetaPALS Zeta Potential Analyzer (Brookhaven Instruments Corporation), after dilution to 50 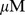 in sterile filtered HEPES buffer. In all experiments the liposome diameter was 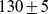 nm with PDI 0.1.

The lipid concentration in liposome suspensions after extrusion was determined by phosphorous analysis, as previously described by Rouser et al. [31]. Briefly, the phospholipid sample was degraded with heat and perchloric acid, reacted with ammonium molybdate and reduced by ascorbic acid. Absorbance of the resulting molybdenum oxides (molybdenum blue) was measured at 812 nm, and lipid sample concentration was determined from a phosphate standard row. All samples were measured in triplicates. A typical final concentration after extrusion at 25 mM was 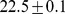 mM.

### Tryptophan fluorescence measurements

#### Peptide self-association

Fluorescence measurements were carried out using an OLIS SLM8000 fluorescence spectrometer equipped with excitation and emission monochromators and polarizers. Tryptophan was excited at 280 nm and emission scans were aquired from 300 to 400 nm with 2 nm step size, 2 s integration time and slit widths 16 nm. Quartz cuvettes with an excitation pathway of 10 mm and an emission pathway of 4 mm were used with 1 mL sample volume. Peptide samples in the concentration range 0.5–100 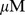 were prepared directly in the cuvette from 100 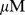 stock solutions in sterile filtered HEPES buffer. Temperature was maintained at 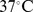 via an external water bath, and the solutions were mixed by magnet stirring in the cuvette.

The recorded spectra were fitted to obtain the peak position and intensity, as described by Burstein et al. [32], using an iterative least-squares fit (built-in in Gnuplot). Self-association of the peptides causes a blue-shift and increase in tryptophan maximum fluorescence, due to increased hydrophobicity near the tryptophan residue, and the blue-shift is used as an indicator of self-association.

#### Liposome partitioning

Fluorescence measurements were conducted similarly to self-association experiments, with integration time 4 s, slit widths 8 nm (excitation) and 16 nm (emission) and polarizers set to 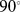 (excitation) and 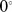 (emission).

The peptide solution was prepared directly in the cuvette and was subsequently titrated with a 20 mM liposome suspension. The peptide was used at a concentration (2 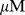) below its self-association concentration, to avoid any signal from self-association.

The fluorescence was initially measured as full wavelength scans (300–400 nm), where the wavelength yielding the largest change with liposome addition was identified (344 nm). Subsequently, the fluorescence was measured as time-scans at 344 nm, and the fluorescence after each liposome addition measured for 5 minutes (increased and stabilized rapidly) and averaged over the last 3 minutes. It was verified that the fluorescence did not change substantially after the 5 minute measurement (up to 1.5 h).

### Liposome partitioning model

The membrane partitioning model is described in detail by Etzerodt et al. [33]. Briefly, the tryptophan fluorescence after the 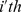 addition of liposome (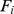) depends on the lipid concentration after the 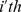 addition (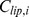) and the partition coefficient (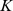) through:
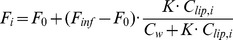
(1)where 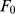 is the initial fluorescence of peptide alone, 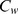 is the concentration of water (55 M) and 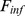 is the fluorescence at ‘full’ partitioning, i.e. after infinite liposome addition, which is a fitted value.

eq. 1 is reorganized to yield
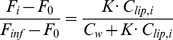
(2)which is equal to the concentration of peptide bound in the membrane 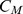 divided by the total concentration of peptide 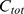, i.e. the fraction of peptide that is membrane-bound. eq. 2 was used for plotting (the left hand side) and fitting (the right hand side), where the fit was an iterative least-squares fit with respect to 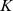 and 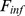 (Gnuplot).

The partition coefficient 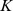 can be converted to the standard Gibbs free energy of partitioning (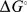) through:
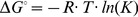
(3)where 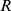 is the gas constant (8.314 J/(K mol)) and 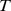 is the temperature (310 K). For simplicity, the absolute value of 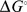 is displayed in graphs.

The partition coefficient 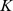 is unitless, but can be converted to the more commonly used molar partition coefficient (in units 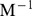) through dividing by the molarity of water (55 M).

Addition of liposomes to the peptide solution causes scattering of excitation and emission light, which yields artifacts in the experimental data that are accounted for by Ladokhin et al. [34]. To minimize these effects the fluorescence was measured using cross-polarized light settings, where the excitation light was horizontally polarized and the emission was measured vertically polarized. This ensures that only the emitted light is measured, as scattering does not alter the polarization, whereas fluorescence randomizes the polarization. However, scattering still causes a decrease in measured fluorescence, which is corrected for by a correction factor 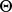, measured by titrating the non-partitioning probe L-Trp with liposomes:
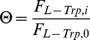
(4)where 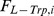 is the fluorescence of L-Trp after the 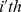 addition of liposome and 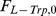 is the initial fluorescence before liposome addition.


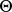
 was fitted to a polynomial, which was used to correct the measured peptide fluorescence during titration with liposomes.

All fluorescence measurements were normalized for peptide concentration, including the slight concentration decrease during titration.

### Interaction with cells

#### Luciferase assay

A BHK cell line (GLP-2R BHK) was previously modified to stably express the human GLP-2 receptor, which controls the expression of firefly luciferase [26], [27].

GLP-2R cells were cultured in DMEM with 10% FCS, 100 U penicillin, 100 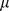
g/mL streptomycin, 1 mM Na-Pyruvate, 250 nM methotrexate, 1 mg/ml geneticin and 0.4 mg/ml hygromycin. The assay was performed in DMEM without phenol red, containing 10 mM HEPES, 1% glutamax and 1 mg/ml OVA.

For experiments, cells were seeded in 96-well plates at 20.000 cells/well (100 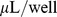) and incubated overnight. The medium was removed and the cells were washed once and replenished with 50 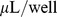 assay medium. The test solution (containing peptide) was diluted in HBSS buffer (containing 10 mM HEPES and 1 mg/ml OVA, pH 7.4) and 50 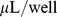 was added to the cells. The cells were incubated for 3 h (at 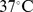 and 5% 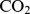) and the test compound was removed. 100 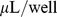 HBSS buffer was added along with 100 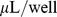 luciferase substrate, the plate was sealed and incubated at room temperature for 30 minutes. The luminescence (Relative Luminescence Units, 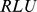) was measured in a topcounter (Packard Topcount) and depends on the peptide concentration as:
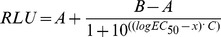
(5)where 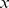 is log(concentration) of the peptide in M, and 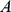, 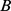, 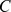 and 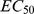 are fitting parameters [35].

The peptide test solutions were diluted to fall within the dynamic range of the assay (approximately 1–100 pM), and on each plate with test solutions a peptide standard row was included, which was fitted according to eq. 5 (using GraphPad Prism).

#### Caco-2 cells

Caco-2 cells (passage 40–65) were cultured routinely [11] in DMEM with 10% (v/v) FBS, 1% (v/v) nonessential amino acids, 100 U penicillin, 0.1 mg/mL streptomycin and 2 mM L-glutamine. The Caco-2 cells were seeded at a density of 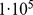 cells/well on 12 well Transwells plates and grown for 14–16 days in DMEM with media change every second day. The cell layer was confirmed to consist of a single monolayer of cells by fixing and staining the cell layer, and visualizing by fluorescence microscopy.

Before transport experiments, DMEM medium was changed to HBSS buffer (containing 10 mM HEPES and 0.1% (w/v) OVA; 0.4 mL apical side and 1 mL basolateral) and left to equilibrate for 60 minutes. Buffer was replaced apically by 0.4 mL test solution at time zero, and the plates were incubated at 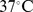 and 5% 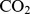 with gentle shaking. Test solutions contained 100 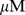 peptide and 0.8 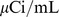 [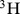]mannitol (a permeability marker). Basolateral samples of 200 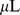 were taken every 15 minutes for 1 hour, and replaced by buffer. The apical test solution and basolateral samples were diluted and analyzed for peptide content with the luciferase assay and for [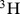]mannitol content in a scintillation counter (Packard TopCount), after mixing 1∶1 with scintillation fluid.

The integrity of cell monolayers before, during, and after experiments was verified by measuring the the translocation of radioactively labelled mannitol and the transepithelial electrical resistance (TEER), using a Milicell ERS-2 epithelial volt-ohm meter (Millipore, USA) [11]. Mannitol translocation and TEER values were not affected by addition of peptide or analogues compared to buffer.

After experiments, the cells were washed twice with buffer and replenished with medium for 24 hour recovery, or washed thrice with ice cold buffer and frozen at 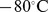 for cell binding and uptake analysis.

For cell binding and uptake studies, all preparations were carried out on ice, and all the buffers were ice cold. The filters were cut out of the inserts and placed in 12 well plates with the cell layer facing up. 250 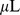 of buffer was added and the cells were scraped off carefully with a cell scraper. The scraping was repeated, and the cell suspensions were pooled and centrifuged (13.000 rpm, 20 min, 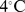). The supernatant was analyzed for peptide content (cell uptake), and the pellet was resuspended in buffer and vortexed thoroughly. The membrane-bound peptide was recovered from the cell debris by addition of ethanol, followed by thorough vortexing and centrifugation (13.000 rpm, 20 min, 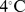). The supernatant solvent was evaporated under nitrogen-flow, and the dry peptide was dissolved in buffer and analyzed for peptide content (cell membrane binding). The cell uptake and membrane binding of peptide and analogues are displayed as the total amount of peptide in the cell layer. It was tested whether the recovery of peptide from cell debris was efficient and/or dependent on acyl chain length. Peptide or analogues were added to the cell debris pellet after the first centrifugation, taken from control cell layers with no added peptide, and after following the subsequent steps for membrane binding, the peptide recovery was measured. Essentially all added peptide was recovered, and there was no measurable difference for the different analogues.

The Caco-2 translocation of peptide or mannitol over Caco-2 layers is expressed as the apparent permeability (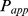), given by:
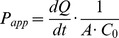
(6)where 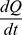 is the steady-state flux of peptide (pmol/s), 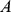 is the surface area of the cell monolayer (1.12 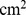), and 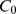 is the initial sample concentration added to the cell layer [11].

### Data analysis

Statistical analysis was carried out using GraphPad Prism, where unpaired Students t-tests were used for comparison and significant differences required p<0.05.

## Results and Discussion

### Characterization of peptide analogues

**Figure 2:**
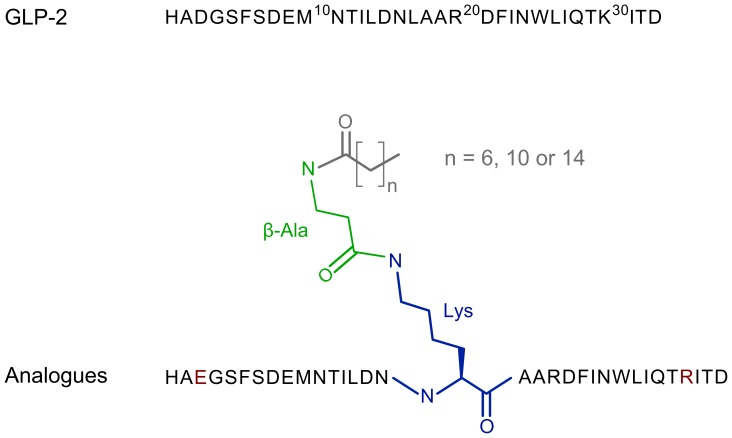
Schematic representation of native GLP-2 and its acylated analogues. GLP-2 is acylated with c8, c12 or c16-chains (grey) at the 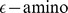 group of Lys17 (blue) via a 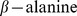 spacer (green). The lysine residue replaces a leucine at position 17 and the natural lysine at position 30 in GLP-2 is mutated to an arginine (red). The aspartic acid at position 3 is mutated to a glutamic acid (red) to avoid racemization during synthesis. None of these mutations caused measurable loss-of-function.

We synthesized and investigated native GLP-2 and the acylated analogues shown in fig. 2, where the acyl chain is conjugated to the 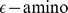 group of a lysine via a 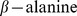 spacer [8], [36].

**Figure 3:**
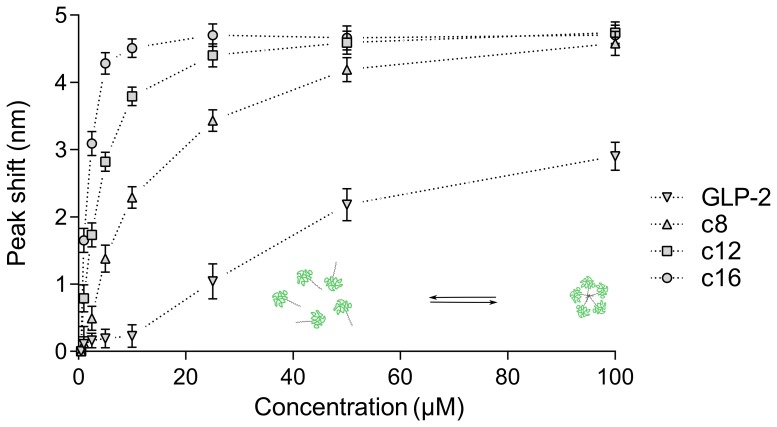
Concentration-dependent self-association of GLP-2 and its analogues. Increasing concentration leads to a blue-shift and increase in tryptophan maximum fluorescence, indicating self-association of the peptides. Control experiments with L-Trp, which does not self-associate, displayed no blue-shift. For the acylated analogues the self-association concentration decreases with increasing chain length, consistent with increased hydrophobicity which renders self-association more favorable. Native GLP-2 is less prone to self-association, and higher concentrations than the displayed 100 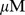 are required to reach full self-association. Data points are mean 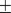 SD of 2 separate experiments, and lines are provided to guide the eye. The sketch serves as an illustration of self-association.

The concentration-dependent self-association of GLP-2 and its analogues was investigated by tryptophan fluorescence (fig. 3). The acyl chains confer increased hydrophobicity, causing self-assembly at lower concentrations for the acylated analogues compared to the native peptide. Furthermore, the self-association concentration decreased with increasing acyl chain length, consistent with increasing hydrophobicity.

At physiological ionic strength, the secondary structure of the c16 acylated analogue was similar to native GLP-2 (investigated by circular dichroism (CD), see fig. S1). It is worth noting that the buffer ionic strength is crucial for self-association, as no self-association was observed with 0 or 10 mM NaCl. This is consistent with electrostatic screening of the peptides's multiple negative charges as a requirement for self-association. The peptide oligomers of native GLP-2 and its c16-analogue were characterized by static and dynamic light scattering (DLS/SLS) and transmission electron microscopy (TEM), which showed that the oligomers were spherical and well-defined in size (smaller for the native peptide than the analogue), and composed of less than 10 monomers (see fig. S2).

### Interaction with model lipid membranes

**Figure 4:**
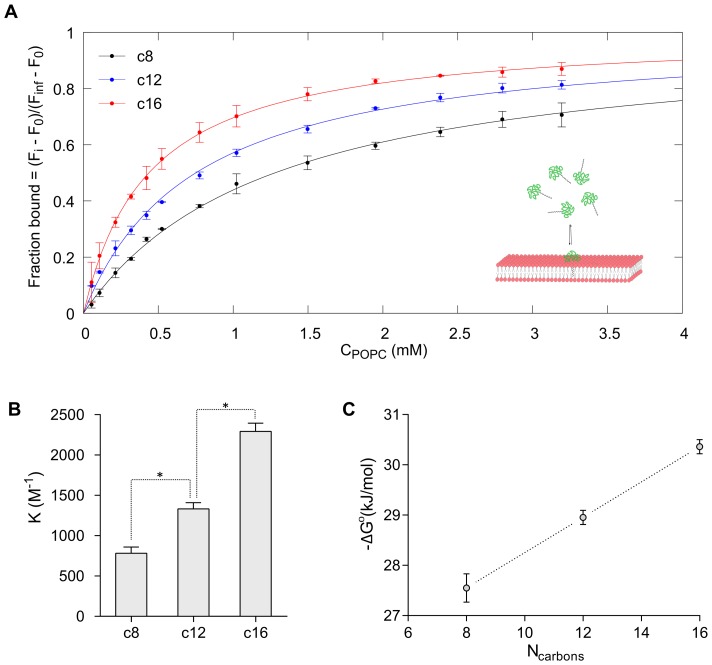
Binding to neutral liposomes. Addition of POPC liposomes to 2 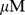 peptide solutions causes an increase in tryptophan fluorescence, indicating partitioning of the peptides into the hydrophobic part of the lipid membrane. A) The peptide fluorescence during titration with POPC liposomes, fitted according to eq. 2 (solid lines), which yields the partitioning coefficients (K) shown in B). For the acylated analogues the partitioning coefficient increases with increasing chain length, and converting to the standard Gibbs free energy (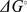) according to eq. 3 yields a linear relationship with the chain length, C). Native GLP-2 partitions very weekly into POPC liposomes, with values below the measurable limit (K<500 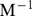), and GLP-2 is therefore not included in the figure. Data points represent mean 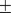 SD of 2 separate experiments and stars in B) indicate significantly different values (p<0.05). The sketch serves as an illustration of membrane-partitioning.

The partitioning of peptides into liposomes was quantified by tryptophan fluorescence, in order to investigate the effect of acylation on interactions with lipid membranes. The data presented in fig. 4 shows that acylation of GLP-2 causes increased partitioning into POPC membranes, where partitioning of native GLP-2 is below the measurable limit and is not included in the graphs. The standard Gibbs free energy of partitioning increases linearly with acyl chain length for the investigated c8, c12 and c16-chains, which is consistent with previous reports of acylated glycine [37]. It is worth noting that the slope of this linear relationship is lower for the GLP-2 analogues than for the acylated glycine analogues, as reported by Peitzsch et al. [37]. This indicates that the dependence of membrane affinity on acyl chain length is influenced by the peptide or amino acid backbone, and that the small glycine residue is more sensitive to chain length than the larger GLP-2 peptide.

It should be noted that the peptides are used at a very low concentration (2 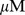) in order to avoid self-association, and that the peptide/lipid ratio is quite low. For subsequent cell experiments (and possible therapeutic applications) the peptides are used at higher concentrations, and the lipid concentration is not well-defined. Therefore, caution should be exerted when comparing liposome and cell results, and speculating on *in vivo* applications.

### Receptor activation

**Figure 5:**
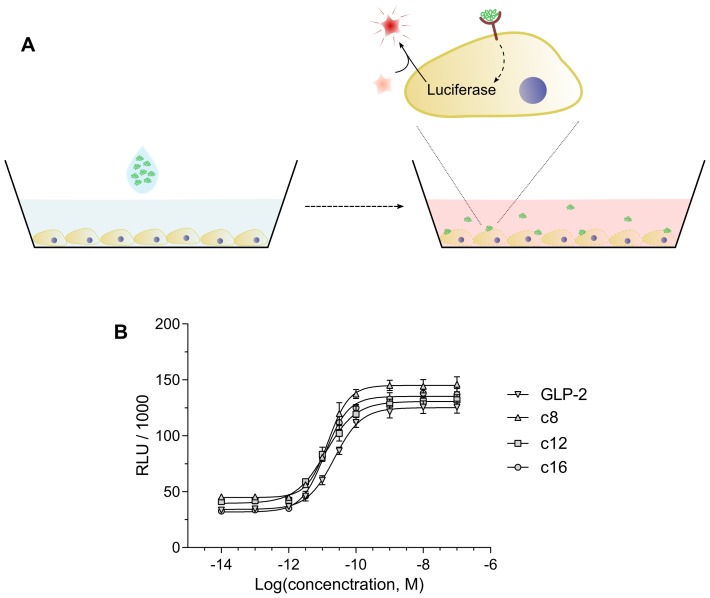
Schematic illustration of the Luciferase receptor activation assay and typical standard curves. A) In order to confirm biological activity of the analogues and measure picomolar concentrations of the peptides, a cell-line with the GLP-2-receptor and a luciferase reporter gene was employed. Upon receptor activation by GLP-2 or its analogues, the cells produce luciferase, which can cleave the substrate luciferin to a luminescent product. B) Representative standard curves of receptor activation (RLU) with increasing peptide concentration, for native GLP-2 and its analogues. Data points represent mean 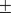 SD from 3 determinations, and solid lines are fitted according to eq. 5.

The biological activity of the analogues was verified in a Luciferase assay (sketched in fig. 5), using a cell line stably expressing the GLP-2 receptor and a luciferase gene, which is transcribed upon receptor activation.

Alteration of therapeutic peptides is accompanied by a risk of reducing potency, and acylation is no exception. However, through a rational choice of acylation site and type and/or screening different acylations, it is often possible to limit the deleterious effects on biological activity [36]. All of of the investigated acylated GLP-2 analogues display similar function compared to native GLP-2, suggesting that these acylations did not negatively impact receptor binding and activation.

The assay is employed to measure picomolar concentrations of GLP-2 and its analogues after *in vitro* experiments with Caco-2 cells, using peptide standard curves fitted according to eq. 5. Thus, the advantage of this cell based reporter assay is its very high sensitivity.

### Interaction with Caco-2 cells

**Figure 6:**
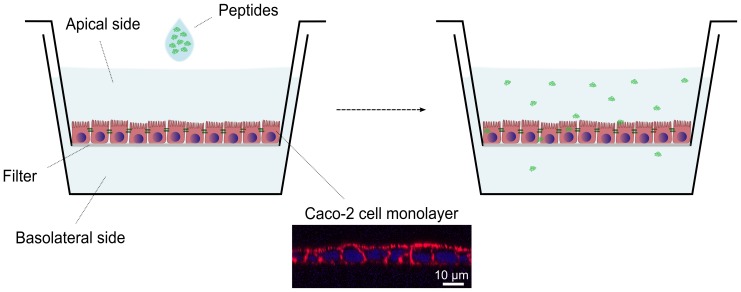
Schematic illustration of the Caco-2 setup. The Caco-2 intestinal model is composed of Caco-2 cells grown in a monolayer on a semipermeable filter support that separates two solution chambers. The peptide of interest is added to the top chamber (modeling the apical side of the intestine, i.e. the intestinal lumen), and the translocated peptide is sampled from the lower chamber over time (modeling the basolateral side of the intestine). Subsequently, the cells are analyzed for peptide content, both in the aqueous parts of the cells (uptake) and in the lipid membranes. The microscopy image in the insert shows a Caco-2 monolayer on the filter support, after fixing and staining for cell nucleus (blue) and actin (red).

The Caco-2 setup is sketched in fig. 6. The translocation of peptide over time from the apical (upper) chamber to the basolateral (lower) chamber was measured, along with the amount of peptide associated with the cells after an experiment, both in the aqueous parts of the cells (uptake) and in the lipid membranes.

#### Peptide translocation

**Figure 7:**
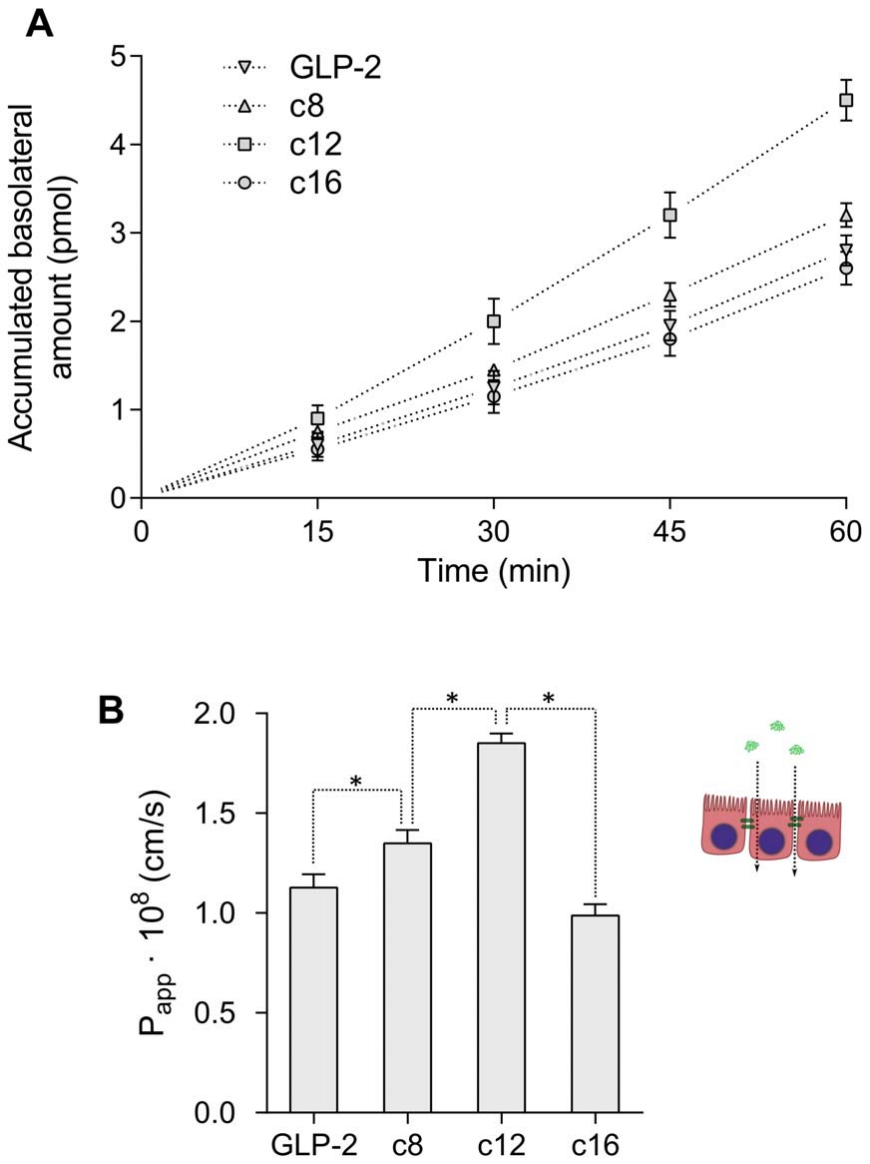
Translocation of GLP-2 and its analogues across Caco-2 cell monolayers. A) Accumulated amount of peptide in the basolateral chamber during the 1 hour experiment. B) Apparent permeability (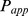) of the peptides, calculated according to eq. 6. Data points represent mean 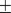 SD of 3 separate experiments, each with 4 repeats, and stars indicate significant differences (p<0.05).

The translocation of GLP-2 and its analogues is presented in fig. 7, where part A shows the accumulated amount of peptide in the basolateral compartment during the 1 hour experiment and part B shows the apparent permeability (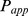), calculated according to eq. 6.

The translocation depends non-trivially on the acylation chain length, as the short and medium chains (c8 and c12) increase the translocation relative to native GLP-2, but the long chain (c16) decreases it slightly. This indicates an optimum chain length where the translocation is increased by increased hydrophobicity and intermediate membrane binding, whereas the long chain causes too effective membrane insertion and strong binding, which limits translocation. For the c12 analogue, the increase in translocation through acylation is roughly a factor 1.5 compared to native GLP-2.

An alternative explanation for the decreased translocation of c16 could be increased self-association, which may limit paracellular translocation through the concomitant increase in size. The extent to which the cellular environment affects self-association is currently unknown, as is the exact degree of peptide translocation through either the paracellular or transcellular route.

The acyl chain length may have an effect on albumin binding, and thereby circulation time, which could limit the useful acyl chain lengths to medium and long chain [8], [36], but this is not fully established [4] and has not been investigated in this study.

#### Cell membrane binding and uptake

**Figure 8:**
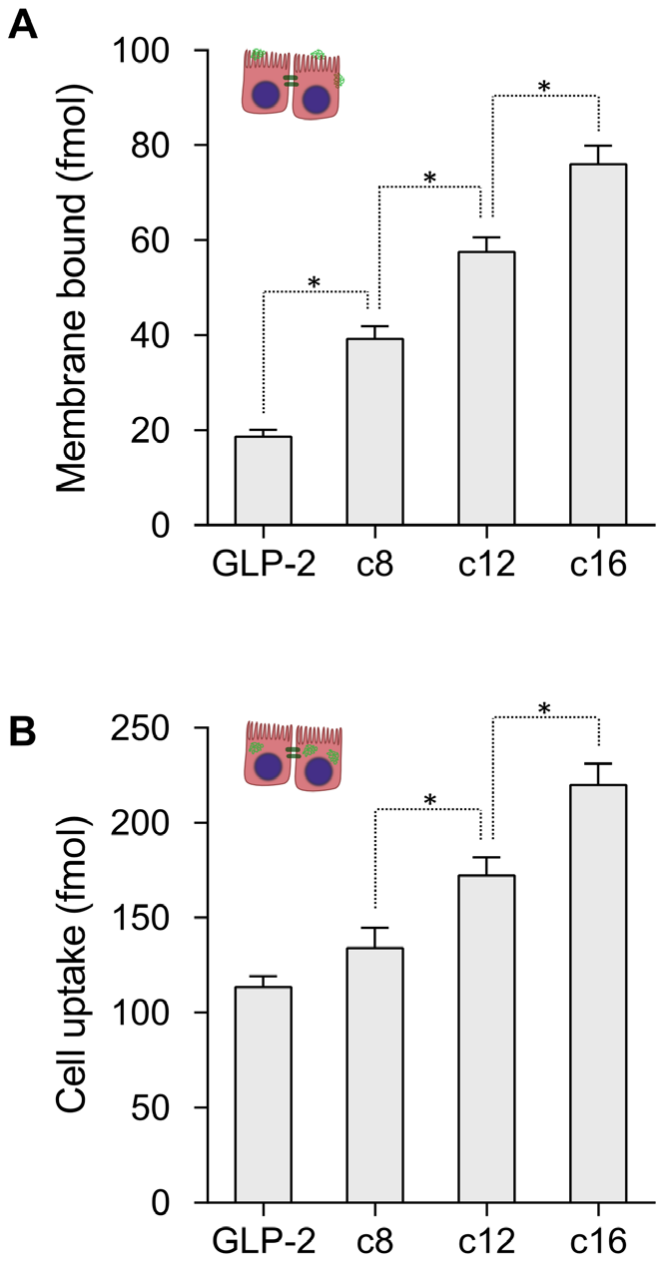
Cell membrane binding and uptake. The amount of peptide bound in the cell membrane A) and the amount of peptide in the aqueous parts of the cell B) increase with acylation length, indicating a larger interaction with cells. For the acylated analogues the membrane binding is roughly linear with chain length, and resembles the observed binding to model membranes shown in fig. 4C. Data points represent mean 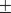 SD of 3 separate experiments, each with 4 repeats, and stars indicate significantly different values (p<0.05).

The cell membrane binding and uptake of GLP-2 and its analogues is presented in fig. 8, where it is evident that both increase with acylation and increasing chain length. The cell membrane binding qualitatively resembles the liposome membrane binding presented in fig. 4C, showing that the chain-length dependence for binding to neutral model membranes is a valid predictor for cell membrane affinity, despite the added complexity of the biological environments compared to a simplified model membrane. It should be noted that cell studies employ a higher concentration of peptide than liposome binding studies, which may cause differences in peptide self-association behavior.

We speculate that the increase in cell uptake with increasing acyl chain length is caused by increased cell membrane binding, where peptide bound to the cell membrane is subsequently taken up more readily.

The observed cell binding qualitatively supports the hypothesis that the decrease in cell translocation for long chain acylation is caused by their increased membrane interactions. The decrease in translocation for c16-acylation compared to c12-acylation is larger than expected from the increased membrane binding, however, this may be explained by the added effect of increased uptake that could lead to intracellular sequestration.

If the membrane binding is a powerful determinant for cell translocation, the liposome partitioning coefficient may be used as a predictor hereof, but this should be investigated in further detail, using other peptides or acylations.

#### Absorption enhancers

The two absorption enhancers EGTA (paracellular) and SDS (transcellular) were employed to assess the effect of different enhancement mechanisms on the acyl chain length dependence.

We hypothesize that paracellular enhancers will have little effect on the acyl chain length dependence, i.e. enhance transport of the peptide and all its analogues to a similar extent, whereas transcellular enhancers that fluidize the membrane may have an increased effect on the long-chain acylated analogue by altering the membrane affinity and/or dynamics of membrane insertion and detachment.

**Figure 9:**
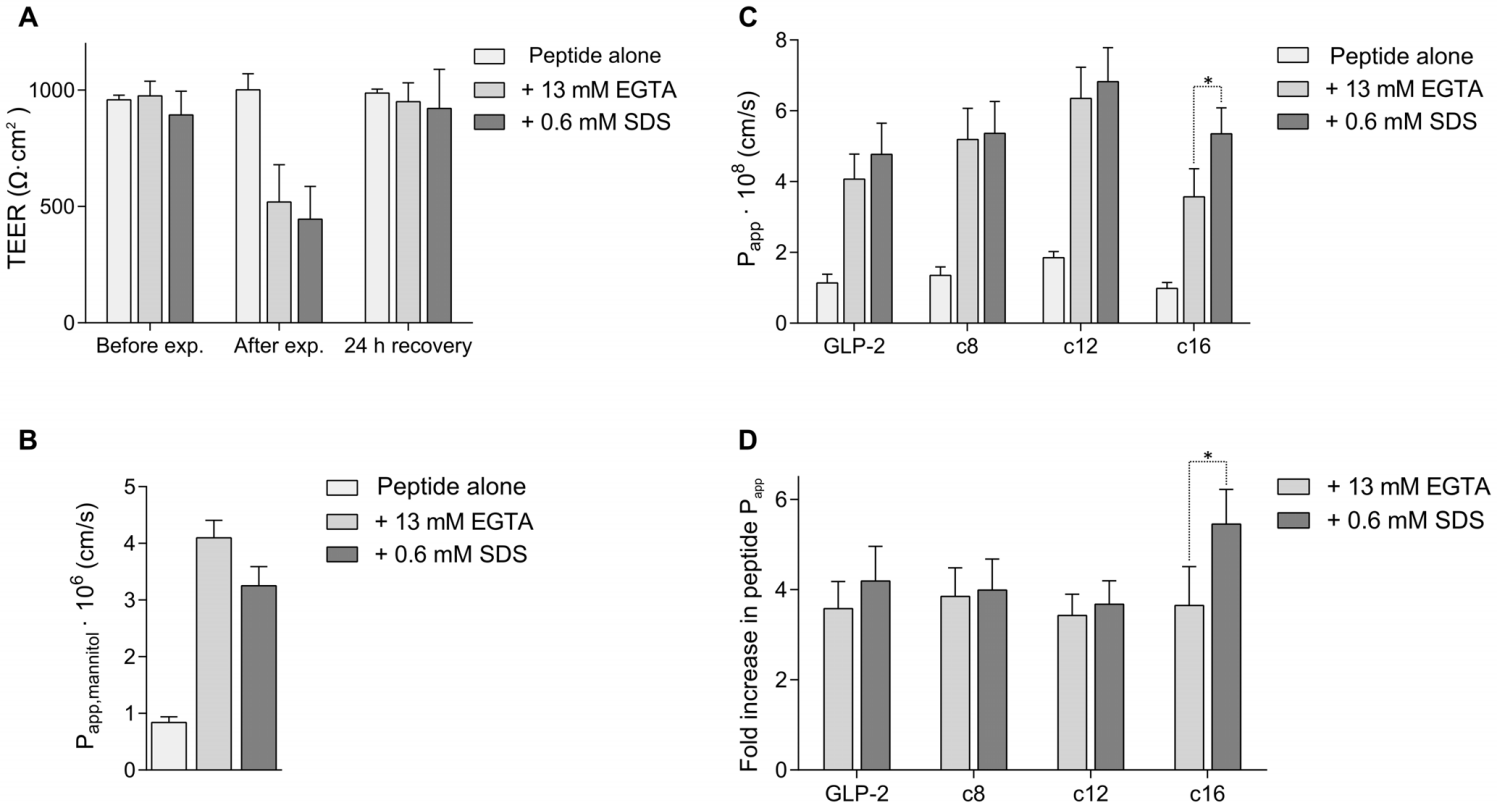
Effect of absorption enhancers on translocation of GLP-2 and its analogues. A) TEER values for Caco-2 cell monolayers after experiments with absorption enhancers EGTA (paracellular) or SDS (transcellular), and after 24 hour recovery in cell medium. B) Mannitol permeability in the presence of EGTA or SDS. C) Peptide permeability in the presence of EGTA or SDS. D) Fold increase in peptide permeability with enhancer, compared to each peptide alone. All data points represent mean 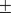 SD of 2 separate experiments, each with 4 repeats, and stars indicate significant differences (p<0.05).

A previously performed dose-response experiment was used to determine the appropriate concentration of absorption enhancers, that yielded increased mannitol transport and a reversible decrease in TEER with full recovery after 24 hours, and the results using the optimal concentrations are presented in fig. 9A and B. The translocation of GLP-2 and its analogues in the presence of EGTA or SDS is presented in fig. 9C. For each peptide, the increased transport in the presence of enhancer was compared to the transport of the peptide alone as shown in fig. 9D, which emphasizes the differences between the two types of enhancer. For EGTA the increase is similar for peptide and analogues, and the dependence on acyl chain length is retained, whereas for SDS the increase is greater for the c16-acylation. These results support the hypothesis that the fluidization of cell membranes caused by SDS are beneficial for the long chain acylation, possibly due to altered membrane insertion. This could be verified by investigating liposome partitioning or cell membrane binding in the presence of SDS. However, despite the added benefit of SDS for the c16-acylation, the translocation of the c16 peptide remains lower than for the c12 peptide, suggesting that the acylation length is the primary determining factor for optimizing translocation. EGTA and SDS are employed as representatives of paracellular and transcellular enhancers, and other enhancers of these types are expected to elicit similar effects.

In conclusion, EGTA and SDS at the concentrations employed increase the translocation while retaining the acyl chain length dependence, with a medium chain length (c12) yielding the highest translocation.

## Conclusions and Perspectivation

We have synthesized and investigated a systematic series of acylated GLP-2 analogues, in order to establish how the membrane binding correlates to *in vitro* intestinal permeability. We find that increasing acyl chain length causes increased self-association and binding to lipid and cell membranes, whereas translocation across intestinal cells displays a non-linear dependence on chain length. Short and medium chains improve translocation compared to the native peptide, but long chain acylation does not. We explain this correlation by an initial benefit for translocation for shorter chains through increased interaction with the cell membrane, which reverts to a hindrance for long chains, i.e. the analogues get stuck in the cell membrane. Measurements of liposome binding may be used to predict the optimal acylation chain length (e.g. the partitioning coefficient of approximately 1300 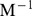 for c12-GLP-2), which can be further investigated by using other peptides or acylation types.

The translocation of peptide and analogues increases in the presence of both paracellular and transcellular absorption enhancers. The acyl chain length dependence persists for the paracellular enhancer and partially for the transcellular enhancer, with an increased benefit for the long chain acylation. This can be explained on the basis of a membrane fluidizing effect of the transcellular enhancer, which alters the dynamics and strength of membrane insertion, however, this requires further investigation. For both enhancers the medium chain acylation (c12) yields the highest translocation, approximately 1.5 times the native peptide.

The presented results suggest that rational acylation of GLP-2 increases intestinal absorption, and may benefit the oral delivery route. However, this should be verified *in vivo*, *e.g.* by investigating the intestinal absorption in an animal model following in situ administration to the intestine. Dosing directly to the rat intestine circumvents the esophagus and stomach, where the peptide would most likely require further stabilization to remain intact, *e.g.* through encapsulation and/or enteric coating.

In order to assert whether the effect of acylation on GLP-2 is general and can be used to benefit oral delivery of other therapeutic peptides, we are currently investigating other acylated peptides. GLP-2 exerts is therapeutic function locally in the intestine, which eases oral delivery and limits the challenging requirement for long circulation times in the blood stream. However, other therapeutic peptides that function systemically or at sites apart from the intestine may benefit thrice from acylation, *i.e.* on intestinal absorption, enzymatic stability, and circulation life time.

## Supporting Information

Figure S1
**Secondary structure of native and acylated GLP-2.** Circular dichroism spectra of GLP-2 and its c16 analogue in buffers with different ionic strength (0–150 mM NaCl). The secondary structure of native GLP-2 and its acylated c16 analogue is different at low ionic strength (as previously reported in [38]), but similar at physiological ionic strength. It should be noted that self-association alters the secondary structure, and as described in the main text, the self-association behavior is affected by acylation. The employed concentration 150 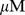 was chosen in order to compare to [38], and at this concentration the peptides are expected to be self-associated.(TIF)

Figure S2
**Size of selected peptide oligomers.** TEM (middle) and DLS (bottom) show that the native peptide and its c16-analogue forms oligomers with radius 2.4 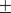 0.1 nm and 2.8 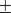 0.1 nm, respectively. SLS-measurements show that the native peptide oligomers are composed of approximately 4 peptide monomers, whereas the c16 analogue oligomers are larger and composed of around 7–10 monomers. The top sketch serves as an illustration of peptide oligomers.(TIF)

Materials S1 **Materials and Methods for Cicular Dichroism (CD), Dynamic and Static Light Scattering (DLS/SLS) and Transmission Electron Microscopy (TEM).**
(PDF)
