# Supplementary figures and images for: Acylation of Glucagon-Like Peptide-2: Interaction with Lipid Membranes and In Vitro Intestinal Permeability
Source: PLoS One. 2014 Oct 8;9(10):e109939. doi: 10.1371/journal.pone.0109939 (PMC4190408; doi:10.1371/journal.pone.0109939)

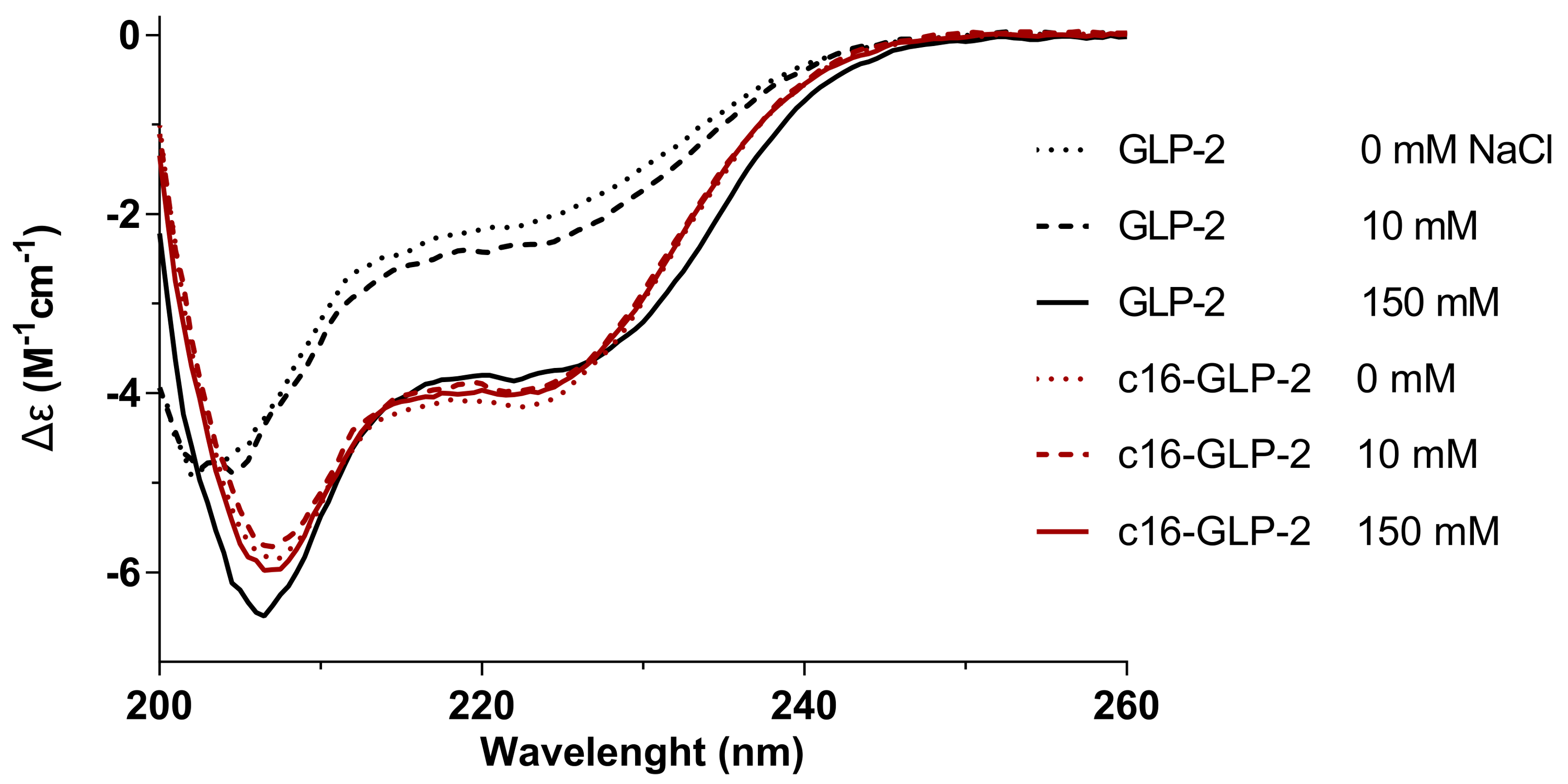

Supplement: Figure S1 — Secondary structure of native and acylated GLP-2. Circular dichroism spectra of GLP-2 and its c16 analogue in buffers with different ionic strength (0–150 mM NaCl). The secondary structure of native GLP-2 and its acylated c16 analogue is different at low ionic strength (as previously reported in [38]), but similar at physiological ionic strength. It should be noted that self-association alters the secondary structure, and as described in the main text, the self-association behavior is affected by acylation. The employed concentration 150 was chosen in order to compare to [38], and at this concentration the peptides are expected to be self-associated. (TIF) [file pone.0109939.s001.tif]

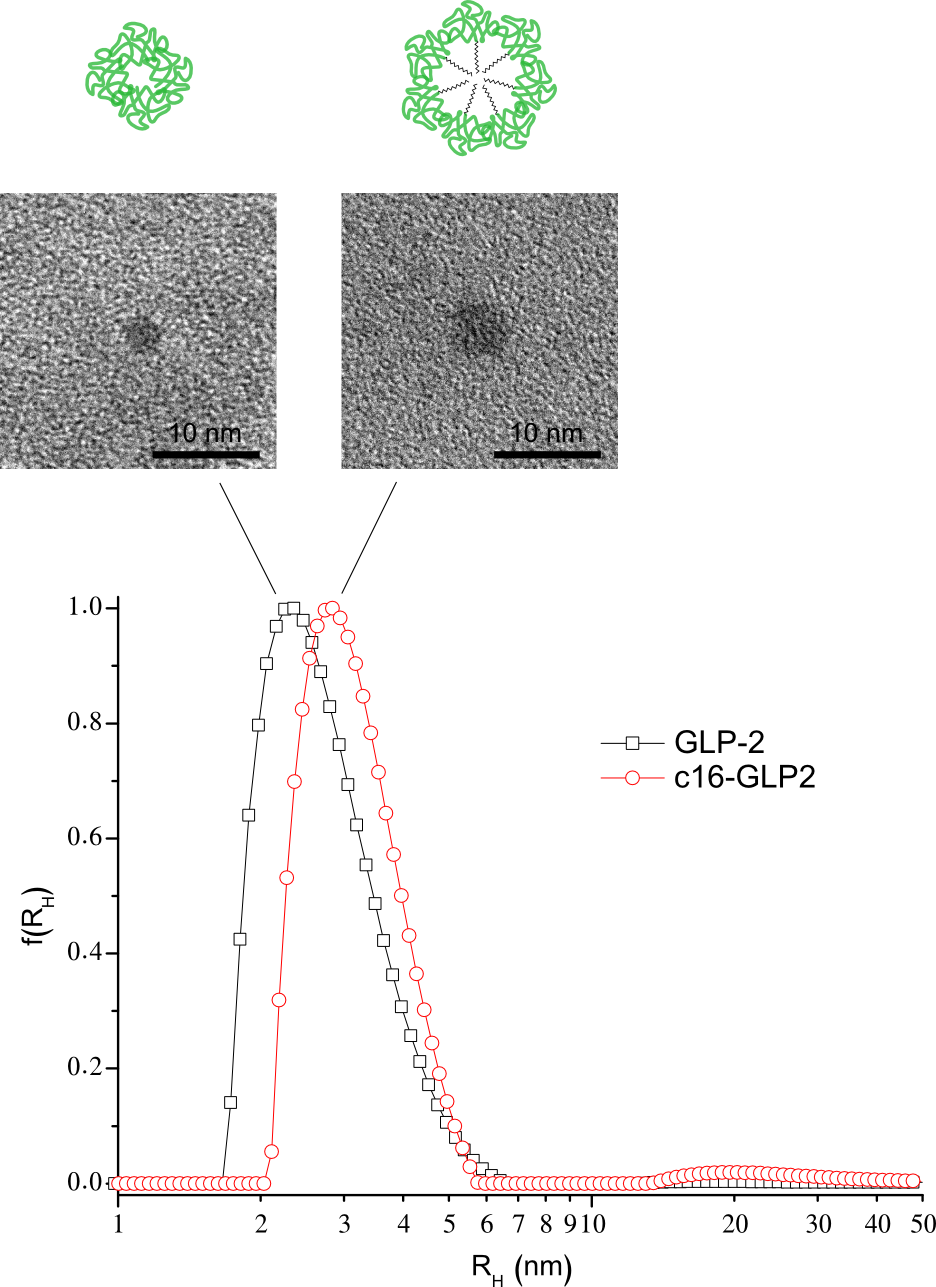

Supplement: Figure S2 — Size of selected peptide oligomers. TEM (middle) and DLS (bottom) show that the native peptide and its c16-analogue forms oligomers with radius 2.4 0.1 nm and 2.8 0.1 nm, respectively. SLS-measurements show that the native peptide oligomers are composed of approximately 4 peptide monomers, whereas the c16 analogue oligomers are larger and composed of around 7–10 monomers. The top sketch serves as an illustration of peptide oligomers. (TIF) [file pone.0109939.s002.tif]
