# Supplementary material for: Acylation of Glucagon-Like Peptide-2: Interaction with Lipid Membranes and In Vitro Intestinal Permeability
Source: PLoS One. 2014 Oct 8;9(10):e109939. doi: 10.1371/journal.pone.0109939 (PMC4190408; doi:10.1371/journal.pone.0109939)
Supplement: Materials S1 — Materials and Methods for Cicular Dichroism (CD), Dynamic and Static Light Scattering (DLS/SLS) and Transmission Electron Microscopy (TEM). (PDF) [file pone.0109939.s003.pdf]

Supporting Materials and Methods

Circular Dichroism, CD

UV CD spectra were obtained on a Jasco-J-815 circular dichroism spectrophotometer (Jasco, Tokyo, Japan). 150 μM peptide samples in HEPES buffers (10 mM HEPES, 0-150 mM NaCl, pH 7.4) were scanned at room temperature in 0.1 mm cells from 200-260 nm using a bandwidth of 2 nm, a response time of 2 s, a data pitch of 0.5 nm and a scanning speed of 50 nm/min. The instrument was calibrated with camphersulfonic acid. A background spectrum of the buffer was subtracted from each peptide spectrum, and the spectra were converted to molar CD using instrument software.

Dynamic and Static Light Scattering, DLS and SLS

A modified commercial Laser Light Scattering (LLS) spectrometer (ALV/DLS/SLS-5022F) equipped with a multi$-\tau$ digital time correlator (ALV5000) and a cylindrical 22 mW He-Ne laser (λ_0 632.8 nm) was used for static and dynamic light scattering (SLS and DLS). LLS was performed at angles between 20° and 150° and the DLS time correlation was analyzed by ALV Correlator 3.0 software. SLS results were analyzed manually, and the molecular weight of the peptide oligomers was obtained using the previously reported partial refractive index of 0.186 mL/g (for proteins in saline buffer with λ_0 633 nm) [1].

250 μM peptide samples in HEPES buffer were filtered through 0.22 μM PVDF filters (non-protein binding Whatman, GE healthcare), while care was taken to avoid dust in the solution and measuring container.

Transmission Electron Microscopy, TEM

TEM imaging was performed on 250 μM peptide samples, as prepared for LLS measurements. 10 μL of sample was applied onto a carbon covered copper grid for 1 minute, after which excess sample was removed carefully with filter paper. The peptides were stained with 3% (w/v) phosphotungstic acid (PTA), by applying 10 μL PTA solution to the copper grid for 2 minutes, after which excess solution was removed. The copper grid was rinsed once with a droplet of MQ water and dried in air for 5 minutes. Samples were imaged at 120 kV in a Technai TEM (FEI).

References

[1] Wen J, Arakawa T (2000) Refractive index of proteins in aqueous sodium chloride. Anal Biochem 280: 327–9.
